# Prior-night sleep duration and the relationship to quality of life and educational context in Norwegian pre-adolescents

**DOI:** 10.1186/s12887-026-06830-6

**Published:** 2026-04-14

**Authors:** Erik Grasaas, Sergej Ostojic, Tonje Holte Stea, Øyvind Sandbakk

**Affiliations:** 1 Teacher Education Unit, University of Agder, Kristiansand, 422, 4604 Norway; 2 Faculty of Health Sciences, University of Pécs, Pécs, Hungary; 3 Faculty of Sport and Health Sciences, University of Agder, Kristiansand, Norway; 4 School of Sport Science, the Artic University of Norway, UiT, Tromsø, Norway

**Keywords:** Sleep recommendations, Life Satisfaction, Child, Schools, Quality of Life

## Abstract

**Purpose:**

Sufficient sleep is associated with improved development, health, and quality of life (QoL). The aim of this paper is twofold: (1) to describe prior-night sleep duration according to sleep recommendations across socioeconomic status (SES), age, gender and screen time use among Norwegian pre-adolescents and (2) to explore the association between reported prior-night sleep duration with QoL and school factors stratified by gender.

**Methods:**

Cross-sectional data was aggregated from the Norwegian Ungdata Junior Survey, collected in 2024. In total, 45,185 children aged between 10 and 12 years provided self-reported data anonymously by filling out an electronic/online questionnaire during school hours.

**Results:**

About half of the study sample (53.1%) reported adherence to international sleep recommendations for children aged 10 to 12 years, with somewhat higher adherence among boys than girls(54.5% vs. 51.8%, p < 0.01). Results from binary regressions revealed positive associations related to QoL and school factors among children adhering to sleep recommendations compared to ones not adhering, with adjusted odds ratio ranging from 1.33 to 1.79 (all p < 0.01).

**Conclusions:**

In this population-based sample of Norwegian children aged 10 to 12 years, approximately half reported not achieving the recommended 9 to 12 h of sleep per night. Reporting prior-night sleep duration in accordance to the international sleep recommendations were strongly associated with higher QoL and positive school related factors among boys and girls, but particularly strong among girls. These findings emphasize the vital role of adequate sleep among pre-adolescents for QoL and optimizing school contexts, offering valuable insight for future practice and policy.

**Supplementary Information:**

The online version contains supplementary material available at 10.1186/s12887-026-06830-6.

## Abstract (plain language summary)

1. Why is this study needed?

Children aged 10 to 12 years are recommended to obtain 9 to 12 h of sleep per day to support optimal health and development. However, global evidence indicates that between one-third to one-half of children report failing to meet these recommendations.

2. What is the key problem/issue/question this manuscript addresses?

The aims of this paper were twofold: (1) to describe prior-night sleep duration according to the sleep recommendation across age, socioeconomic levels, gender and screen time use among Norwegian children aged 10 to 12 years and (2) to explore the association between reported prior-night sleep duration with quality of life and school factors stratified by gender.

3. What is the main point of your study?

Research specifically examining sleep adherence and quality of life in Norwegian pre-adolescents is scarce. This study addresses that gap by providing novel data from a large population-based Norwegian sample, offering insight into potential cultural and age-specific differences.

4. What are your main results and what do they mean?

This research identifies associations between children’s prior-night sleep duration and indicators of quality of life in an educational context, offering insights that may inform practice and policy development by highlighting the potential importance of sleep for daily functioning and well-being among children in Norway.

**Supplementary Information:**

The online version contains supplementary material available at 10.1186/s12887-026-06830-6.

## Introduction

According to the consensus statement of the American Academy of Sleep Medicine, children aged 10 to 12 years are recommended to obtain 9 to 12 h of sleep per day to support optimal health and development [1]. However, global evidence indicates that between one-third to one-half of children report failing to meet these recommendations [2–5]. Multiple factors have been shown to impact sleep adherence in children, including socioeconomic factors [4], physical activity levels [6], screen time use [7] and chronic health conditions [8]. Although reported sleep duration represents a relatively simple and one-dimensional measure, sleep problems are typically more complex and multidimensional, encompassing sleep quality, sleep timing, nocturnal disturbances and daytime fatigue; nevertheless, sleep duration remains as a robust predictor of children’s health [9].

A systematic review by Chaput and colleagues [10], including more than 500,000 children and youths, demonstrated that shorter sleep duration was associated with adverse physical and mental health outcomes. Quality of life (QoL) has been shown to represent a natural and meaningful mental health outcome, which captures children’s overall physical, emotional and social aspects of life [11] and thereby provides insight into the everyday life and children’s subjective well-being. Furthermore, previous research has revealed strong positive associations between longer sleep duration and subjective well-being among children [10, 12], however too long sleep duration is associated with adverse outcomes in some contexts [13]. Hence, the relationship between sleep and quality of life may vary across cultural contexts [14] and between younger (10 years) and older (12 years) children [15]. Examining how sleep duration relates to QoL among Norwegian children across these ages contributes to novel evidence to a field largely dominated by studies from non-Nordic settings.

Adequate sleep is related to having the energy required for development, learning and engagement in daily life [16]. In this context, school plays a pivotal role in childhood, serving as a primary setting where children spend time, energy and acquire foundational skills. Results from previous research has indicated that longer sleep duration are associated with higher academic performance [16–18], school engagement [19] and cognitive functioning [20], while factors such as gender differences and socioeconomic status (SES) warrant consideration [19]. Findings among US children have revealed that children adhering to sleep guidelines were more likely to value school performance, complete homework, and achieve better academic outcomes [21]. Nevertheless, this relationship is likely multifactorial, and such behaviours cannot be attributed to sleep patterns alone but may also reflect broader contextual and confounding influences. Furthermore, maintaining a regular bedtime has shown to significantly increase the likelihood of meeting sleep recommendations [4], suggesting that time spent in bed correlates with actual sleep among older children. However, as older children more often gain access to smartphones, this represents a particularly noteworthy period; because, for early adolescents, it is common for more than an hour to pass between going to bed and actually falling asleep [22, 23]. Reducing screen time before bedtime is therefore recommended for youth to minimize possible harmful effects related to sleep and well-being [23–25].

It is therefore essential to examine the potential consequences of insufficient sleep among Norwegian pre-adolescents aged 10 to 12 years. Although substantial research has documented links between sleep duration, subjective well-being, and school factors, notable gaps remain. Research specifically examining sleep duration and QoL in Norwegian pre-adolescents is scarce. This study addresses that gap by providing novel data from a large population-based Norwegian sample, offering insight into potential cultural and age-specific differences, alongside careful consideration of covariates, such as SES, gender, age and screen time use. This research can yield valuable insights for both practice and policy development, by uncovering favourable associations between prior-night sleep duration relative to the sleep recommendations and daily functioning and well-being among children in Norway. Hence, the aims of this paper were twofold: (1) to describe prior-night sleep duration according to the sleep recommendation across SES, age, gender and screen time use among Norwegian children aged 10 to 12 years and (2) to explore the association between reported prior-night sleep duration with QoL and school factors stratified by gender.

## Methods

### Data collection and the Ungdata Junior study

Data collection was administered by the Norwegian counties and their respective municipalities. The municipalities contacts the schools for distribution of the survey. Each school allocated one school hour to conduct the survey electronically. The teacher was present during the survey, and for those children who chose not to participate in the survey, they received alternative academic tasks. Ungdata serves as a valuable population-based dataset for planning and implementing public health interventions in Norway [26] and was financed by grants from the Norwegian Directorate of Health.

Ungdata Junior is a population-based annual survey in Norway. The Norwegian Social Research at Oslo Metropolitan University, in collaboration with the Regional Centre for Drug Rehabilitation and the Municipalities sector’s organization administer the study. The dataset is regarded as representative, as data is collected across all of Norway. Since 2017, Ungdata Junior has gathered responses from over 200,000 participants between the ages of 10 and 12 years. For detailed information about the motivation, development and use of Ungdata Junior, please see the protocol of Løvgren & Jacobsen [27].

### Study design and participants

This cross-sectional study used data from the 2024 Ungdata Junior survey, which included 45,185 Norwegian children aged 10–12 years. Ungdata Junior study entails several demographic and health-related variables and all data is anonymous. This current dataset does not possess any data on school or municipality affiliation due to privacy guidelines and general data protection rules.

### Study variables

#### Prior-night sleep duration

Prior-night sleep duration was assessed using the question “*How many hours of sleep did you get last night?”.* Seven response alternatives were provided for the participants, ranging from 6 h or less, and hourly categorized up to 12 h or more. These response alternatives were recoded into a dichotomous variable to determine whether the prior-night sleep duration was in accordance with the international sleep recommendations (9 h to 12 h) or not (8 h or less) [1].

#### Quality of life

The assessment of quality of life derived from five aspects of the children’s everyday life. Herein, the respondents were asked how satisfied they were with their: (1) Parents, (2) Friends, (3) School, (4) Living area and (5) Own health. Four response categories were provided as alternatives: *“not satisfied at all”*,* “a little satisfied”*,* “quite satisfied”* and *“very satisfied”.* The first three response alternatives were recoded into lower satisfaction, and the latter category recoded into high satisfaction. These QoL aspects, have been used throughout the years for the original Ungdata survey (for adolescents) and in Ungdata Junior (for children) but are not formally validated.

#### School factors

School factors were assessed by six statements regarding satisfaction with school-related events. The statements included how the children perceived; (1) School satisfaction, (2) Teacher care, (3) Sense of belonging, (4) Free time in school, (5) Dread of going to school and (6) Get bored during school hours. All statements had four response alternatives: *“totally agree”*,* “somewhat agree”*,* “somewhat disagree”*, and *“totally disagree”.* The first response alternative was recoded into high satisfaction, and the latter three categories were recoded into lower satisfaction with school factors. These questions have been administered among serval hundreds of thousands of participants in the original Ungdata survey (for adolescents) and in Ungdata Junior but are not formally validated.

#### Covariates

Relevant covariates were age (grade level), SES and screen time use. Grade level was used as a proxy for age and was assessed by using the question *“Which grade level are you in?”.* Responders were provided with three response alternatives: *“5th grade”*,* “6th grade”* and *“7th grade”.* SES comprised of four questions of prosperity, included whether their family possessed a car, with response alternatives *“No”*,* “Yes*,* one”* or *“Yes*,* two or more”*. Having your own bedroom, with response alternatives: *“Yes”* or *“No”.* Number of times travelling abroad, with response alternatives *“None”*,* “Once”*,* “Twice”* or *“More than twice”.* And the question of how many iPads/computers are in your home, with response alternatives: *“None”*,* “One”*,* “Two”*,* “Three”* or *“More than three”.* Screen time use was assessed with the question *“Approximately how many hours do you spend in total on activities in front of a screen?”*,* seven* response alternatives were provided: *“no time”*,* “less than an hour”*,* “one to two hours”*,* “two to three hours”*,* “three to four hours”*,* “four to six hours”* and *“more than six hours”*.

### Ethical consideration

Approximately 2 weeks in advance of the survey, both parents and children were informed of the Ungdata Junior study. Participation was voluntary, and the children were free to skip any questions and withdraw any time without any reason. Parents/guardians received written information about the study’s objective and that they may reserve their child from participation. This recruitment procedure is approved by the Norwegian Agency for Shared Services in Education and Research (ref. 821474), known as SIKT [28]. For the majority of children, the survey questions are not perceived as problematic to answer. However, to mitigate potential discomfort, Ungdata has implemented measures to reduce any inconvenience experienced by participants. There measures include informing that both parents and pupils may contact the school health service or the Red Cross helpline should they wish to talk to an adult following participation [29]. The Ungdata Junior study is in accordance with the Helsinki Declaration, and all questions are approved by SIKT. This study has followed the Strengthening the Reporting of Observational Studies in Epidemiology (STROBE) guidelines for transparent and systematic reporting (Supplementary Table 1) [30].

### Statistical analyses

All statistical analyses were performed using IBM SPSS Statistics for Mac, Version 25.0 (IBM Corp., Armonk, NY, USA). Descriptive measures for continuous variables are reported as means and standard deviation (SD). Categorical variables are presented as counts and percentages. Chi-square test was administered to compare differences between gender within categorical variables. Sleep duration was dichotomized into adherence to the international sleep recommendation (9 h to 12 h) [1] or not adhering (8 h or less). The outcome variables, QoL and school factors were dichotomized to distinguish respondents reporting high satisfaction with aspects of life and school from those reporting lower levels of satisfaction. This approach was chosen to capture sleep adherence’s impact on presence versus absence of satisfaction and to account for a pronounced ceiling effect in the distribution. Logistic regressions were conducted to explore the relationship between adherence to sleep recommendations or not (independent variable) on QoL (dependent variable) and school factors (dependent variable) by adjusting for gender, SES, grade level and screen time use. Regression models were presented by the total sample and stratified by gender, with odds ratio (OR), confident intervals (CIs) and p-values, whereof values of < 0.05 were considered statistically significant. Due to the high response rate in this current study and large population-based sample, bootstrapping nor imputation was deemed necessary.

## Results

### Participants

In total, 45,185 Norwegian boys and girls aged 10 to 12 years reported prior-night sleep duration and were included in this study. The current study sample had an equal distribution of girls 50.1% (*n* = 22,653) and boys 49.9% (*n* = 22,532). A total of 32.2% (*n* = 13,922) derived from 5th grade, 33.4% (*n* = 14,540) from 6th grade and 34.4% (*n* = 14,978) from 7th grade. The response rate among study variables were high, > 97.8% for all QoL variables, and > 96.9% for all variables related to school factors. Response rate for all study variables is available as Supplementary Table 2.

### Descriptive statistics

About half of the study sample (53.1%) reported prior-night sleep duration in accordance with the international sleep recommendations for children aged 10 to 12 years (Table 1), with somewhat higher compliance among boys than girls (54.5% vs. 51.8%, *p* < 0.01). Boys reported consistently higher satisfaction than girls across all QoL aspects, with the largest discrepancy disclosed for satisfaction with own heath (50.7% vs. 41.6%, respectively). About half of the sample reported high agreement of school satisfaction, perceived teacher care, sense of belonging and enjoyment of the free time in school. More boys (34.8%) than girls (26.7%) reported high level of boredom during school hours (*p* < 0.01).

**Table 1:** Characteristics of study sample

Study variables	All, *n* (%) Girls, *n* (%) Boys, *n* (%)		
Prior-night sleep duration according to recommendations	24,006 (53.1%)	11,727 (51.8%)	12,279 (54.5%) **
Quality of life			
High satisfaction with parents	34,329 (73.0%)	16,945 (72.4%)	17,384 (73.8%) **
High satisfaction with friends	25,077 (53.3%)	11,712 (50.1%)	13,365 (56.8%) **
High satisfaction with school	15,111 (32.1%)	7,346 (31.4%)	7,765 (33.0%) **
High satisfaction with living area	23,608 (50.2%)	11,219 (48.0%)	12,389 (52.6%) **
High satisfaction with own health	21,685 (46.1%)	9,742 (41.6%)	11,943 (50.7%) **
School factors			
High school satisfaction	23,338 (49.6%)	11,473 (49.0%)	11,865 (50.4%) **
High perception of teacher care	25,240 (53.7%)	12,738 (54.5%)	12,502 (53.1%) **
High sense of belonging	22,454 (47.7%)	9,670 (41.3%)	12,784 (54.3%) **
High enjoyment of free time in school	22,849 (48.6%)	9,852 (42.1%)	12,997 (55.2%) **
High degree of school-related dread	4,507 (9.6%)	2,064 (8.8%)	2,443 (10.4%) **
High level of boredom during school hour	14,436 (30.8%)	6,243 (26.7%)	8,193 (34.8%) **

Chi square tests were used to test gender differences, * *p* < 0.05, ** *p* < 0.01

The reported prior-night sleep duration according to sleep recommendations progressively decreased by grade levels (Table 2), from 64.2% in 5th grade to 40.7% in 7th grade. Marginal differences were uncovered between boys and girls in 5th and 6th grade level, however only 36.1% of girls in 7th grade complied to sleep recommendations, compared to 45.1% among boys (*p* < 0.01). The same patterns were unveiled for screen time use, whereas little screen time use up to excessive use progressively decreased prior-night sleep duration according to sleep recommendations for both boys and girls, revealing that about only a quarter of girls (26.3%) having 6 h or more of daily screen time use complied to sleep recommendations. Results for descriptive analyses revealed no clear observed trends between SES variables and compliance to sleep recommendations. Nevertheless, it is noteworthy that a higher proportion reported prior-night sleep duration according to recommendations among those without having their own bedroom (56.4%) compared to the ones having their own (52.8%). Additionally, reporting a greater number of cars or more than three iPads/computers did not coincide with better compliance; rather, more moderate and restrained household resources coincided with higher compliance with sleep recommendations.

**Table 2:** Characteristics of prior-night sleep duration according to sleep recommendations across grade level, screen time use and socioeconomic status

Study variables	Prior-night sleep duration according to sleep recommendations		
Grade level	All (N %)	Girls (N %)	Boys (N %)
5th grade	8984 (64.2)	4694 (66.3)	4260 (62.2) **
6th grade	8039 (55.3)	3903 (53.8)	4104 (56.9) **
7th grade	6095 (40.7)	2654 (36.1)	3421 (45.1) **
Screen time			
No screen time	300 (60.1)	133 (60.2)	167 (60.1)
Less than one hour	1732 (68.7)	1133 (70.7)	599 (65.1) **
1 to 2 h	5802 (66.6)	3411 (66.0)	2391 (67.4)
2 to 3 h	6077 (57.5)	3121 (55.5)	2956 (59.9) **
3 to 4 h	5018 (49.6)	2117 (44.8)	2901 (54.0) **
4 to 6 h	3004 (41.1)	1080 (34.9)	1924 (45.8) **
6 h or more	1514 (33.7)	451 (26.3)	1063 (38.5) **
Socioeconomic status			
Family owns a car			
No	753 (54.7%)	388 (56.4%)	365 (53.1%) *
Yes, one	6,636 (53.3%)	3,315 (51.6%)	3,321 (55.3%) **
Yes, two or more	16,504 (52.9%)	7,982 (51.7%)	8,522 (54.2%) **
Having your own bedroom			
Yes	22,125 (52.8%)	10,795 (51.4%)	11,330 (54.3%) **
No	1,644 (56.4%)	837 (56.0%)	807 (56.7%)
Times travelling with your family			
None	3,959 (51.4%)	1,941 (50.9%)	2018 (52.2%)
Once	8,765 (53.2%)	4,311 (51.7%)	4454 (54.8%) **
Twice	5,946 (53.6%)	2,945 (52.4%)	3001 (55.1%) **
More than twice	4.978 (53.5%)	2,353 (51.8%)	2625 (55.4%) **
Computers/iPads in your home			
None	146 (49.8%)	68 (48.6%)	78 (51.3%)
One	815 (52.3%)	444 (51.1%)	371 (53.8%)
Two	2,154 (54.1%)	1,175 (53.1%)	979 (55.6%)
Three	2.989 (54.1%)	1,601 (52.4%)	1,388 (56.3%) **
More than three	17,418 (52.8%)	8,203 (51.4%)	9,215 (54.2%) **

Chi square test for gender differences, *p < 0.05, **p < 0.01

### Regressions analyses

Results from regressions revealed that prior-night sleep duration that complied with sleep recommendation rather than not complying were positively associated with all aspects of QoL, with odds ratio (OR) ranging from 1.33 to 1.79 (Table 3, all *p* < 0.01). Across the QoL domains, girls reported stronger association between QoL and prior-night sleep duration, which complied to sleep recommendations than boys. Specifically, prior-night sleep duration according to sleep recommendations was associated with increased odds of being satisfied with their own health among both girls [OR = 2.00; 95% CI (1.88–2.12)] and boys [OR = 1.58; 95% CI (1.49–1.68)].

**Table 3:** Binary logistic regressions between prior night’s sleep according to recommendations or not (independent variable) and high or low satisfaction with quality of life (dependent variables)

Total sample	Parents	Friends	School	Living area	Own health
	OR (95% CI)	OR (95% CI)	OR (95% CI)	OR (95% CI)	OR (95% CI)
Not complying (ref)	1	1	1	1	1
Sleep adherence	1.71 (1.63–1.80) **	1.33 (1.28–1.39) **	1.64 (1.57–1.72) **	1.52 (1.46–1.59) **	1.79 (1.71–1.86) **
Girls					
Not complying (ref)	1	1	1	1	1
Sleep adherence	1.87 (1.75–2.01) **	1.37 (1.29–1.45) **	1.71 (1.60–1.82) **	1.62 (1.52–1.72) **	2.00 (1.88–2.12) **
Boys					
Not complying (ref)	1	1	1	1	1
Sleep adherence	1.53 (1.42–1.64) **	1.29 (1.21–1.37) **	1.57 (1.47–1.66) **	1.42 (1.34–1.51) **	1.58 (1.49–1.68) **

Adjusted for socioeconomic status, grade level and screen time use, *p < 0.05, **p < 0.01

Binary regressions revealed that prior-night sleep duration according to sleep recommendations compared to ones not complying, revealed higher odds of school satisfaction [OR = 1.60; 95% CI (1.54–1.67)], perceived teacher care ([OR = 1.51; 95% CI (1.44–1.57)], sense of belonging in school [OR = 1.52; 95% CI (1.46–1.58)] and enjoyment during free time in school [OR = 1.35; 95% CI (1.29–1.40)]. Moreover, lower odds were revealed among those reporting prior-night sleep duration according to sleep recommendations compared to those not complying, with lower odds of the dread of going to school [OR = 0.71; 95% CI (0.66–0.76)], and boredom during school hours [OR = 0.63; 95% CI (0.60–0.65)]. Across all school-related factors, girls reported stronger associations to between school factors and prior-night sleep duration according to sleep recommendations than boys (Table 4).

**Table 4:** Binary logistic regressions between prior night’s sleep duration according to sleep recommendations or not and high or low satisfaction with school factors

Total sample	School	Teacher care	Belonging	Free time	Dread of school	Boredom
	OR (95% CI)	OR (95% CI)	OR (95% CI)	OR (95% CI)	OR (95% CI)	OR (95% CI)
Not complying (ref)	1	1	1	1	1	1
Sleep adherence	1.60 (1.54–1.67) **	1.51 (1.44–1.57) **	1.52 (1.46–1.58) **	1.35 (1.29–1.40) **	0.71 (0.66–0.76) **	0.63 (0.60–0.65) **
Girls						
Not complying (ref)	1	1	1	1	1	1
Sleep adherence	1.65(1.55–1.74) **	1.56(1.47–1.65) **	1.60(1.50–1.70) **	1.38(1.30–1.47) **	0.67(0.61–0.75) **	0.61(0.57–0.66) **
Boys						
Not complying (ref)	1	1	1	1	1	1
Sleep adherence	1.55(1.47–1.64) **	1.43(1.35–1.52) **	1.44(1.36–1.52) **	1.31(1.24–1.39) **	0.75(0.68–0.83) **	0.64(0.61–0.68) **

Adjusted for socioeconomic status, grade level and screen time use, *p < 0.05, **p < 0.01

## Discussion

This study described prior-night sleep duration according to the international sleep recommendations across SES, age, gender and screen time use among Norwegian pre-adolescents aged 10 to 12 years and explored the association between prior-night sleep duration with QoL and school factors stratified by gender. The main findings revealed that about half of the study sample reported prior-night sleep duration to not comply with the international sleep recommendations, and that compliance to sleep recommendations declined progressively by grade level and by increasing screen time use. Reporting prior night sleep duration in accordance to sleep recommendations were strongly associated with higher QoL and positive school related factors across gender, but particularly strong among girls.

Our findings revealed that about half the study sample reported prior-night sleep duration not in accordance with the international recommendations of sleep, which aligns with the higher end of previous research evidence.

 [2–5]. Moreover, descriptive results revealed a progressive decline in reported compliance to sleep recommendations across grade levels. Notably approximately two out of three girls in 7th grade did not meet the recommendation of minimum of 9 h of sleep. The high prevalence of insufficient sleep in late childhood may set an early development trajectory that persists throughout adolescence. According to the US National Sleep Foundation, adolescents are recommended to obtain a minimum of 8 h of sleep per night, and existing evidence indicate that the proportion failing to meet these recommendation increases from pre-adolescents to old adolescents [31]. Although prevalence estimates varies considerably across countries (up to 86%) [32], Norwegian adolescents appear to be among those with the highest rates of insufficient sleep, with studies reporting non-adherence to recommendations ranging from 73% to 85% throughout adolescence [33, 34].

Based on previous research in pre-adolescents [35–39], our descriptive findings expectedly revealed that boys reported higher QoL across all dimensions and higher satisfaction in school compared to girls. However, girls reported higher perceived teacher care and lower dread of going to school. These findings may reflect interrelated mechanisms; for instance, higher perceived teacher care may contribute to lower dread of going to school. Previously, younger Norwegian adolescent girls have reported higher perceived teacher care than boys, and evidence suggests that teacher care is positively associated with well-being among younger adolescents [40]. This pattern indicates that gender differences in pre-adolescence may be related to social perceptions and interpersonal dynamics. Girls also tend to score higher than boys on measures of social support and friendship, suggesting that social interactions and friendships are more particularly important for their perceived QoL [41]. Moreover, the number of caring friends has been reported to play a role for well-being among adolescent girls, further underscoring the importance of social connectedness among this group [42].

Among girls with the highest levels of reported screen time use, only a quarter complied to sleep recommendations. Although our data do not capture the timing for screen use, evidence shows that extended screen exposure often occur at the expense of sleep [43]. Pre-adolescents are in a developmental stage characterized by ongoing physical and mental maturation, making them particularly vulnerable to disruptive effects. In particular, the light and sounds from scrolling may interfere with various bodily processes, including physiological responses such as melatonin production, emotional responses such as self-awareness, and other physical responses, for example hunger, thirst, and natural fatigue, all of which can ultimately affect the feeling of sleepiness [44]. Due to these harmful effects of excessive screen use on sleep and wellbeing, limiting screen time, especially in the evening before bedtime, is widely recommended for children and adolescents [23]. High levels of screen time and social media use in early adolescence have further been associated with poorer well-being in later adolescence, particularly among girls [45], which underpin the importance of establishing healthy sleep-related habits in this period of life.

In addition, our findings revealed that more moderate and restrained household resources coincided with higher compliance with sleep recommendations. The proxies for SES used in Ungdata junior primarily reflects material affluence (e.g., number of tablets/iPads) and may therefore overlook important dimensions such as parental education and household routines, which are presumably closely linked to sleep behaviours. Findings have suggested that children and adolescents with higher parental education is associated with more restrictive attitudes towards screen use and meeting screen time recommendations [46]. This suggests that higher SES households do not necessarily provide children with more tablets or iPads; in some cases, they may even have fewer.

Despite the relatively narrow age range in our sample, there might be considerable differences in sleep need between a 10-year-old and a 12-year-old. While our findings revealed that reported prior-night sleep duration in accordance to sleep recommendations was strongly associated with positive school related factors, previous research has suggested that the optimal sleep duration for academic performance among 12-years-olds may fall slightly below the recommended minimum of the 9 h [47]. Although official recommendations shift from a minimum of 9 h to a minimum of 8 h between the ages of 12 and 13, research indicates a more gradual developmental transition, as the sleep duration associated with “academic optimum” in adolescence has been estimated to be approximately 8.5 h per night [48]. Furthermore, studies have shown considerable individual variability in sleep needs, varying between 8.5 h and 11 h among 11-year-olds, which suggests that such differences should be considered when addressing sleep needs, as children with shorter or longer sleeps needs may be differentially affected by standardized sleep recommendations. Regardless of what may constitute as an exact optimal amount of sleep, findings clearly underscore that shorter sleep is linked to lower academic and cognitive performance, and behavioural problems in pre-adolescents [47, 49].

Reported compliance to sleep recommendations seems to be particularly important for girls. Our results indicated consistently stronger associations between prior-night sleep duration according to sleep recommendations and all QoL dimensions among girls compared to boys, with the strongest association observed for girls’ perception of their own health. Although prior research has shown that sleep problems are associated with psychiatric symptoms in both boys and girls across childhood and adolescence, insufficient sleep duration in particular seems to constitute a more specific risk factor for psychological difficulties among girls [50]. Moreover, adequate sleep duration in pre-adolescents is associated with physical changes, including the timing of pubertal events, as findings suggests that children aged > 11 years who sleep less than recommended experiences an earlier menarche [51], which have been linked to poorer long term health, such as higher risk of diseases and all-cause mortality in adulthood [52]. Therefore, following the sleep recommendations in pre-adolescents, seems crucial for their well-being, particularly in girls due to several interconnected factors that impact their physical [53], mental and emotional health [54], and even predict long term health [1].

### Strengths and limitations

Several limitations are warrant for consideration. First, the use of cross-sectional data precludes identification of any causal evidence, and associations may act bi-directional. We have therefore no guarantee of the true direction of the associations. It would be helpful to more explicitly address reverse causation and residual confounding (e.g., mental health, physical activity, family routines), clarifying that lower well-being may also lead to shorter sleep in this cross-sectional design. Hence, there are a strong likelihood of bidirectional relationships between poor mental health, lower quality of life, or school-related stress, which may contribute to shorter or poorer sleep. However, due to the limitation of the study design, temporal ordering of these variables cannot be determined. Second, Ungdata possess a large battery of health-related questions, which are not formally validated. The quality of life (QoL) items used in this study are not derived from a formally validated instrument, despite the use of these questions year after year, which limit the comparability of our findings to other populations and reduces the validity of findings. Regarding socioeconomic status (SES), measures in Ungdata stems from the Family Affluence Scale II, which originates from WHO by Currie and colleagues [55, 56]. However, in the Ungdata junior dataset, there are no measures of parental educational levels, and thereby no validated SES measure, which should be considered as a limitation. Moreover, the use of a single-item self-report measure (screen time and sleep) may limit reliability and content validity, as it is less able to capture the multidimensional nature of screen-based or sleep behaviours and may be more susceptible to random measurement error and reporting bias. Third, we acknowledge that intra-individual variability in sleep (e.g., weekday–weekend differences and random fluctuations) may lead to misclassification and non-differential measurement error, which could attenuate observed associations and limit interpretation of the findings as reflecting habitual sleep. Forth, in the present study, we have chosen to retain the dichotomized variables for several other reasons. Our primary aim was to facilitate interpretability and align the analyses with clinically and practically meaningful thresholds. For example, the categorization of sleep duration was based on established guideline-relevant cut-offs, enabling a clearer distinction between individuals meeting versus not meeting recommended levels. Moreover, dichotomization of presence versus absence of satisfaction is more meaningful and applicable from an educational context, and additionally to account for a pronounced ceiling effect in the distribution, as collapsing categories helped ensure more robust and stable estimates, particularly in categories with low frequencies. Nonetheless, this approach may conceal meaningful variation and should be interpreted with caution. Fifth, despite Ungdata is considered a national representative population-based sample, our findings may not necessarily be generalized to other populations. Fifth, this comprehensive survey may not be easy for all children to answer independently, according to the data material 84.2% reported the questions were easy to answer, indicating a small proportion of participants with potential recall biases or other challenges. Sixth, we acknowledge that the study context may have created an incentive structure where some students chose to participate in order to avoid alternative schoolwork. This could have implications for both participation patterns (selection bias) and response behavior such as random responding (Hawthorne effect). Finally, this current study also holds several factors that add benefits to the credibility of findings, such as thorough and transparent reporting by following the STROBE guidelines, and the use of a representative population-based sample of children aged 10 to 12 years with high response rate across study variables. The rigor procedure of preparing and cleaning data performed by Ungdata, increases the validity and reliability of the findings.

### Implications and future directions

This study demonstrates about half of the participating children reported prior-night sleep duration not in accordance with the recommended minimum of 9 h of sleep per night, and progress even further with increasing age and screen time use. To build upon previous research, our findings show that reported prior-night sleep duration according to sleep recommendations is positively associated with all dimensions of quality of life (QoL) and school-related factors, even after controlling for multiple relevant covariates, in a large, population-based sample of Norwegian pre-adolescents, which underpin valuable implications for practice. Educators, school health services, and caregivers should actively promote healthy sleep habits, and the results suggest that public health and educational initiatives should explicitly incorporate sleep recommendations into the school curriculum, and health promotion strategies. Future research should prioritize longitudinal studies to establish causal relationships and changes over time, explore other sleep-related variables besides sleep duration in pre-adolescents, such as sleep quality, sleep variability and daytime sleepiness to understand a more complex nature of mechanisms. Taken together, these findings indicate that sleep duration is a pivotal lever for enhancing QoL and optimizing school contexts among pre-adolescents.

## Conclusions

In this population-based sample of Norwegian children aged 10 to 12 years, approximately half reported not achieving the recommended 9 to 12 h of sleep per night. Reporting prior-night sleep duration according to the sleep recommendations were strongly associated with higher QoL and positive school related factors among boys and girls, but the relationship was stronger among girls. These findings emphasize the vital role of adequate sleep among pre-adolescents for promoting QoL and optimizing school contexts, including gender-specific differences. Accordingly, this study offers valuable insight for practice and policy, which should tailor strategies to support that children meet the international sleep recommendations, and thereby enhance their QoL, school development, and long-term health, in this pivotal developmental period from childhood to adolescence.

## Supplementary Information

Supplementary Material 1.

Supplementary Material 2.

## Data Availability

Data supporting the results of this study is available upon request from the Norwegian Agency for Shared Services in Education and Research (SIKT) [27]. Reference to dataset from SIKT: NOVA & Bakken, Anders. (2024). Ungdata junior 2024 [10.18712/NSD-NSD3157-V1].

## Abbreviations

QoL: Quality of life
SES: Socioeconomic status
CI: Confidence interval
SD: Standard deviation
SIKT: Norwegian Agency for Shared Services in Education and Research
WHO: World Health Organization
STROBE: Strengthening The Reporting Of Observational Studies
