# Supplementary material for: Prior-night sleep duration and the relationship to quality of life and educational context in Norwegian pre-adolescents
Source: BMC Pediatr. 2026 Apr 14;26:482. doi: 10.1186/s12887-026-06830-6 (PMC13202889; doi:10.1186/s12887-026-06830-6)
Supplement: Supplementary file 2 — Supplementary Material 2. [file 12887_2026_6830_MOESM2_ESM.docx]

**Supplementary table 2:**

Response rate in study variables

| Variable | N | Response rate % |
| --- | --- | --- |
| **Quality of Life**  Parents  Friends  School  Living area  Your health  **School factors**  School satisfaction  Perceived teacher care  Sense of belonging  School hours satisfaction  Dread of going to school  Bored during school hours  Parents  **Socioeconomic status**  Own car(s)  Own bedroom  Travel(s) abroad  How many iPads/computers  **Screen time use**  **Grade level**  **Gender** | 44,065  44,050  44,000  43,766  43,853  45,095  44,845  44,781  44,902  44,182  44,965  44,065  45,185  45,124  44,834  44,634  44,495  43,510  45,185 | 97.5%  97.5%  97.4%  96.9%  97.1%  99.8%  99.2%  99.1%  99.4%  97.8%  99.5%  97.5%  100%  99.9%  99.2%  98.8%  98.5%  96.3%  100% |
